# Characterization of low molecular weight urinary proteins at varying time intervals in type 2 diabetes mellitus and diabetic nephropathy patients

**DOI:** 10.1186/s13098-019-0430-1

**Published:** 2019-05-17

**Authors:** Dhara N. Patel, Kiran Kalia

**Affiliations:** 1 Department of Medical Laboratory Technology, Charotar Institute of Paramedical Sciences, CHARUSAT, CHARUSAT-Campus, Highway 139, Off, Nadiad-Petlad Road, Changa, Gujarat 388421 India; 2 Present Address: National Institute of Pharmaceutical Education and Research (NIPER-G), Gandhinagar Opposite Air Force Station, Palaj, Gandhinagar, Gujarat 382355 India

**Keywords:** Urinary 2DE, Urinary proteomics, Diabetic nephropathy, Diabetic kidney disease

## Abstract

**Background:**

To identify low molecular weight urinary proteins capable of detecting diabetic nephropathy patients which may predict renal alterations at early stages and prevent it from worsening further.

**Method:**

Three hundred ninety (390) age-matched subjects were divided into 8 groups depending upon duration of diabetes and the severity of renal damage. Urinary proteome profile of all subjects was determined with the help of microfluidic array. Participants with similar profile were further selected to study proteome map of urinary low molecular weight proteins with the help of 2 dimensional gel electrophoresis.

**Results:**

Out of 390 total patients 268 patients showed a similar one dimensional proteomic pattern. Further, two-dimensional urinary proteomic pattern of these patients with molecular weight < 50 kDa was studied. Eight proteins with molecular weight 11, 15, 17, 23, 34, 38 and 46 kDa were identified with MALDI-QTOF. These low molecular weight proteins showed gradual increase in urinary excretion along with the duration of diabetes and severity of renal damage.

**Conclusion:**

The study concludes that proteomic analysis might be a useful tool for detecting some novel markers capable of detecting patients susceptible to diabetic nephropathy in the early phase.

**Electronic supplementary material:**

The online version of this article (10.1186/s13098-019-0430-1) contains supplementary material, which is available to authorized users.

## Introduction

Type 2 diabetes mellitus (T2DM) patients are frequently diagnosed with well established microalbuminuric stage due to its inadequate diagnosis and prognosis [1]. The progression of diabetic nephropathy (DN), generally silent and remains clinically unnoticed [2]. DN, if undiagnosed or untreated leads to end-stage renal disease and the patients are solely left with only two survival options which are costly, dialysis or kidney transplantation. The morbidity and mortality rate of DN patients are escalating and there is thus a urgent need for new promising biomarkers capable of diagnosing DN prior to appearance of microalbumin and rise in serum creatinine. This may also lead to suggesting some new strategies for therapeutics.

Urinary proteomics has recently been applied to understand pathophysiology and complexity of pathogenic mechanisms during T2DM induced nephropathy [3, 4]. The proteomic techniques are considered sensitive capable of detecting low abundance proteins. We compared two-dimensional urinary proteomic pattern of proteins with molecular weight < 50 kDa. For detecting the earliest possible LMWP, T2DM patients without any secondary complications (T2DM duration 1–20 years), and T2DM patients with microalbuminuria and nephropathy as a secondary complication were included. Further, the pattern obtained from T2DM patients with and without nephropathy was compared with protein pattern obtained from non-diabetic-nephropathies (NDN) like IgA nephropathy, Focal Segmental Glomerulosclerosis, Minimal Change Disease and Nephrotic Syndrome patients. We assumed that while comparing protein profiles of all study groups, it may be useful in selecting LMWPs that are exclusive for T2DM only. The present study thus was designed to isolate and identify urinary low abundant and low molecular weight proteins (LMWPs) (< 50 kDa) which can predict the possibility of diabetic nephropathy at earliest. It will further be helpful in the pathophysiological and therapeutic interventions for diabetes and diabetic nephropathy.

## Methodology

Patients attending outpatient’s clinic of Muljibhai Patel Urological Hospital, Nadiad, Gujarat, India, between December 2009 and April 2013 were enrolled for the present cross-sectional study. The study plan was approved by the institution’s Ethical Committee. All patients fulfilled; inclusion criteria i.e. age > 25 and estimated GFR (eGFR) > 30 ml/min/1.73 m^2^. Patients with any disorders/diseases, undergone any surgery, past history of consuming any drugs, lactating females and pregnant women were excluded. All T2DM patients were either on insulin, oral hypoglycemic agents (OHA) like metformin/glibenclamide or a combination of both. Each patient provided fasting blood/urine samples. The blood samples were collected in plain as well as EDTA coated pre-sterile vacutainers (BD-Biosciences purchased). Blood was centrifuged at 3000 rpm at 4 °C for the collection of serum/plasma and stored at − 80 °C for further analysis. Clean midstream urine samples were collected in pre-sterile containers (Tarson) and centrifuged at 3000 rpm for 15 min at 4 °C to remove the cellular debris. The aliquots were stored at − 80 °C with protease inhibitors to prevent further protein degradation. Participants were divided into various groups based on the duration of diabetes:

Group I: normal healthy adults (NHA); Group II: T2DM participants with duration of 0–5 years; Group III: T2DM participants with duration of 5–10 years; Group IV: T2DM participants with duration of 10–15 years; Group V: T2DM participants with duration of 15–20 years; Group VI: microalbuminuria with T2DM duration of > 15 years (MIA); Group VII: diabetic nephropathy with T2DM duration of > 15 years (DN); Group VIII: non-diabetic nephropathy (NDN) which includes; IgA nephropathy (N = 22), focal segmental glomerulosclerosis (N = 12), minimal change disease (N = 9), nephrotic syndrome (N = 7).

### Biochemical assay

Serum and urinary creatinine levels were measured by Jaffe’s Kinetic method [5]. Glomerular filtration rate (eGFR) was calculated by Cockroft and Gaults Equation [6]. The Glycated Hemoglobin was quantified by the method of Parker et al. [7]. Fasting blood glucose was estimated based on GOD-POD enzymatic reaction using Automatic Random Access Biochemistry Analyzer XL-300 (Erba Diagnostics, Mannheim). Urinary microalbumin was determined using ELISA-based diagnostic method (Biovendor Research Diagnostics; European Union).

### Microfluidic chip array and 1 dimensional electrophoresis

Urinary total protein was precipitated by acetone method. To 5 ml of the urine sample, 40% ice-cold acetone was added slowly and gradually. The urine-solvent mixture was kept at − 20 °C for 4 h following centrifugation at 14,000×*g* for 20 min to obtain the precipitated protein. The total protein obtained was further quantified by the Bicinchoninic acid method following the instructions manual of QuantiProTM BCA Assay Kit provided by Sigma. The automated capillary electrophoresis was carried out by following instructions according to Protein 230 Kit supplied by Agilent Life Technologies. The assay was performed on Agilent 2100 Bioanalyzer. The sensitivity and quantitative detection limit of the assay are 30–2000 ng/μl protein.

### Sample preparation for 2-dimensional gel electrophoresis (2DE)

Five patients with similar urinary 1 dimensional protein profile from each group were selected for further 2DE study. The 2DE of each selected participants were carried out twice. Low molecular weight proteins (LMWPs) from precipitated proteins were isolated with the help of centrifugal cutoff membranes procured from Merck Millipore. For obtaining LMWPs up to 50 kDa, 100 kDa cutoff followed by a 3 kDa cutoff was applied. The procedure was according to instructions manual of Merck, and the centrifugation was done at 7500×*g* for 45 min at 4 °C. For collecting the protein samples from the cutoff membrane, an inverse spin centrifugal technique was applied. The protein obtained was then dissolved in the rehydration buffer and quantified using BCA method.

### 2-Dimensional gel electrophoresis

For isoelectric focusing an immobilized pH gradient (IPG) strip of pH 3 to 10 and 7 cm in length (BioRad) was loaded with 150 µg protein. The gel strip was rehydrated along with the sample for 14 h at 20 °C. The samples were focused for 5 h up to 10,000-V h. The IPG strip was then equilibrated with dithiothreitol equilibration buffer and iodoacetamide equilibration buffer for 15 min each.

### Second dimension 2D-PAGE

The second dimension was carried out on 8–16% SDS–polyacrylamide pre-casted Tricine gels procured from BioRad. The run was conducted at 10 mAmp for 8 h maintaining 4 °C. A low molecular weight marker was run along with the samples.

### Coomassie blue staining and image analysis

The gels were rinsed with deionized water for 1 min with constant shaking. Further, the gels were transferred to Coomassie blue stain. The gel was left in the staining solution for overnight on the shaker. Next day the gel was transferred to the detaining solution. The gel was destained till the background was clear. Once the gel was destained, it was further analyzed with the image analyzer (BioRad). The image was captured, and the gel spots were compared using Lab Image software (BioRad). The protein spots were then manually cut and stored in deionized water and sent for Q-TOF analysis.

### Protein identification by Q-TOF analysis

Spots isolated from the gel were destained and digested in-gel with trypsin for 24 h at 37 °C. Tryptic peptides were extracted with 0.1% trifluoroacetic acid, purified using Zip-TipC18 pipette tips from Millipore, and analyzed on a 6550 I-Funnel quadrupole time-of-flight mass spectrometer (Q-TOF LC/MS) (Agilent Technologies), connected to a CapLC. An MS–MS survey method was used to acquire MS–MS spectra. Data analysis was performed using the Data Processing Software-Agilent Technologies Spectrum Mill MS Proteomics Workbench (Rev B.04.00.127). Peptide alignment was performed using peptide alignment tool of Mass Hunter, Mill Spectrum (Agilent) connected to Swiss-Prot database. Peptides consisting of five or more amino acids were used and matched to either a non-redundant human IPI or the Swiss-Prot database to identify the corresponding proteins.

### Statistical analysis

Comparisons among groups were performed using ANOVA with Tukey’s posthoc test for multiple comparisons, p values < 0.05 were considered statistically significant. The data are reported as mean ± SD. For analyzing one-dimensional profile, the differences in frequency of all proteins (spectral data) were arbitrarily determined. The presence of a protein was greater than 80% in one group, but less than 20% in the other group was considered significant. The coefficient of variation (CV) was calculated using the formula: %CV) standard deviation/mean × 100%.

## Results

### Anthropometric and clinical data of patients

Table 1 summarizes the details of anthropometric parameters, serum and urinary biochemical parameters of 390 subjects. The participants were distributed in eight different groups on the basis of their duration of diabetes and renal function. Body Mass Index (BMI) increased moderately (p = 0.126) in all the test groups compared to healthy controls. All T2DM patients, with or without nephropathy showed significantly increased fasting blood glucose and glycated hemoglobin in comparison to healthy controls (p < 0.001). Significantly increased serum creatinine and microalbumin levels were observed only in MIA, DN and NDN patients (p < 0.001). Further, a significant decline in eGFR of patients with MIA, DN, and NDN (p < 0.001) was observed. However, microalbumin, serum creatinine, and eGFR were within normal range in T2DM patients with 0–5, 5–10, 10–15 and 15–20 years of diabetes duration.

**Table 1:** Fasting glucose, serum fructosamine, HbA1C, serum and urinary creatinine, eGFR, urinary microalbumin and total protein in control and test groups

Serum and urinary biochemical parameters	Control	Type 2 diabetes (T2DM) with normoalbuminuria				Type 2 diabetes with micro albuminuria	Diabetic nephropathy	Non diabetic nephropathy
		0–5 years	5–10 years	10–15 years	15–20 years			
No. of patients	48	59	38	37	25	50	83	50
BMI (Kg/m^2^)	21.57 ± 0.9	27.21 ± 0.7 ^NS^a	26.52 ± 0.7 ^NS^ab	26.21 ± 0.3 ^NS^abc	26.11 ± 0.5 ^NS^abcd	23.92 ± 0.7 ^NS^abcde	24.01 ± 0.4 ^NS^abcdef	23.62 ± 0.8 ^NS^abcdefg
Fasting glucose mg/dl	93.96 ± 2.1	149.4 ± 7.5 ***a	138.5 ± 7.5 ***a^NS^b	147.4 ± 5.3 ***a^NS^bc	150.2 ± 6.7 ***a^NS^bcd	155.6 ± 4.2 ***a^NS^bcde	157.6 ± 5.5 ***a^NS^bcdef	92.8 ± 3.8 ^NS^a
HbA1C%	4.5 ± 0.3	7.5 ± 1.3 ***a	7.0 ± 0.2 ***a^NS^b	8.6 ± 0.3 ***a^NS^bc	8.9 ± 0.5 ***a^NS^bcd	9.6 ± 0.8 ***a^NS^bcde	10.6 ± 0.8 ***a^NS^bcdef	4.3 ± 0.1 ^NS^a
Serum fructosamine	143.3 ± 7.4	300.6 ± 19.0 ***a	315.6 ± 13.8 ***a b^NS^	354.2 ± 18.6 ***a^NS^bc	428.8 ± 27.5 ***a **bc^NS^d	469.4 ± 17.5 ***abcd^NS^e	523.5 ± 12.4 ***abc**d^NS^ef	158.4 ± 8.06 ^NS^a
Serum creatinine mg/dl	0.75 ± 0.1	0.89 ± 0.5 ^NS^a	0.9 ± 0.04 ^NS^ab	1.1 ± 0.7 ^NS^abc	1.1 ± 0.1 ^NS^abcd	1.7 ± 0.7 ***abc^NS^de	4.3 ± 0.8 ***abcdef	4.9 ± 0.2 *** abcdef^NS^g
eGFR ml/min/1.73 m^2^	94.7 ± 4.3	98.8 ± 4.7 ^NS^a	98.2 ± 5.0 ^NS^ab	96.3 ± 6.4 ^NS^abc	80.0 ± 6.1 ^NS^abcd	63.3 ± 5.3 *** abcd ***e	34.5 ± 1.9 ***abcd **e *f	33.4 ± 4.4 *** abcde^NS^fg
Urinary micro albumin mg/g creatinine	12.1 ± 0.7	12.6 ± 0.9 ^NS^a	10.6 ± 1.1 ^NS^ab	12.45 ± 3.1 ^NS^abc	12.73 ± 1.9 ^NS^abcd	165.1 ± 10.2 ***abcde	322.5 ± 7.8 ***abcdef	329.8 ± 4.5 *** abcdef^NS^g
Urinary total protein by BCA mg/dl urine	9.6 ± 0.78	10.7 ± 0.56 ^NS^a	17.8 ± 0.1 ^NS^ab	18.1 ± 0.34 ^NS^abc	19.4 ± 0.11 ^NS^abcd	186.4 ± 0.96 ***abcde	448.3 ± 0.12 ***abcdef	450.1 ± 0.89 *** abcdef^NS^g
Urinary total protein by microfluidic array mg/dl urine	13.7 ± 0.5	13.8 ± 0.7 ^NS^a	21.3 ± 0.1 ^NS^ab	23.4 ± 0.18 ^NS^abc	26.9 ± 0.27 ^NS^abcd	192.4 ± 1.6 ***abcdef	568.9 ± 0.57 ***abcdef	571.8 ± 0.46 *** abcdef^NS^g

The results are expressed as mean + SE and p < 0.05 is considered significant. * p < 0.05, ** p < 0.001, *** p < 0.0001

a—compared with control, b—compared with 0–5 years T2DM, c—compared with 5–10 years T2DM, d—compared with 1–15 years T2DM, e—compared with 15–20 years T2DM, f—compared with microalbuminuria, g—compared with diabetic nephropathy

NS, non significant

### One dimensional urinary proteomic profile of T2DM patients

**Fig. 1:**
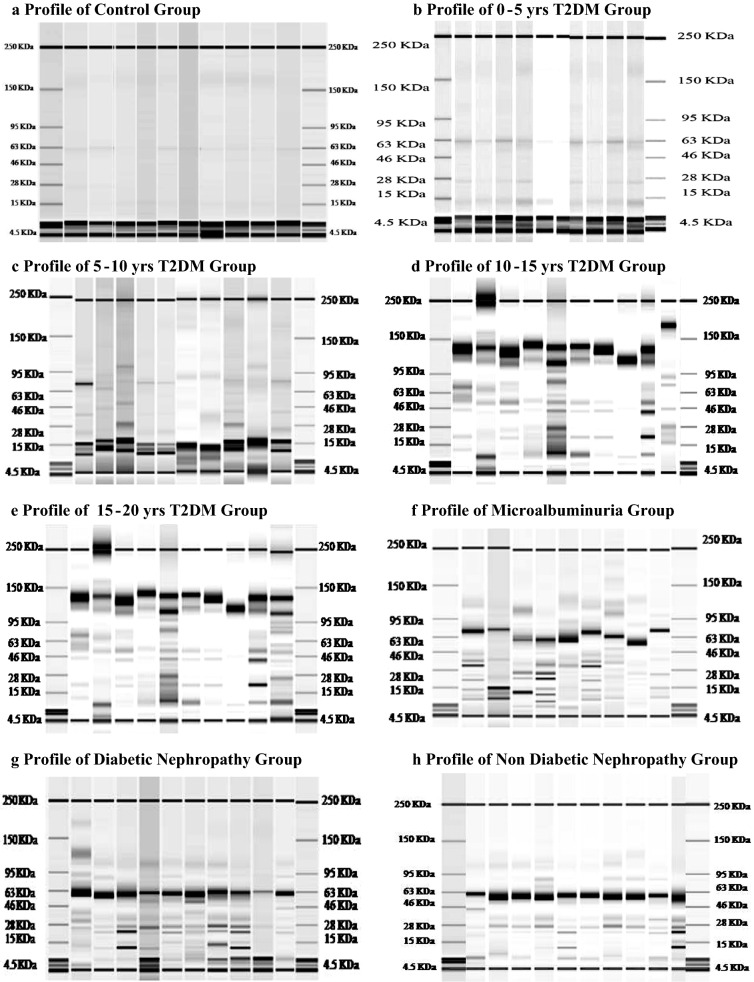
Urinary protein profile of various groups. One dimensional protein profile of 390 participants showing various range of proteins excreted in urine. Total 268 participants showed a similar profile (Additional file 1) and were further selected for 2 dimensional gel electrophoresis study. The present one dimensional profile depicted appearance of low molecular weight proteinuria of clinical significance in early diagnosis of diabetic nephropathy

Proteins from every single urine samples were differentiated in the form of bands depending on their molecular weight with the help of microfluidic array. The protein profile was in the form of one-dimensional SDS-PAGE electropherogram (Fig. 1a–h) (Additional file 1). These data provided a detailed description of concentration and molecular weight of individual fractionated protein. A single protein was detected with its corresponding spectral peak and particular elution time. The peaks of the proteins/peptides were studied for identifying whether the proteins are single or a mixture of protein. A crisp and sharp peak indicated the presence of one single protein/peptide. While a blunt and broad peak indicated the presence of two or mixture of proteins/peptides. Such overlapping peaks were observed due to proteins of similar molecular weight. Thus, such peaks were not considered for further analysis. The selection of candidate proteins was based upon its frequency of appearance. Only those proteins with frequency more than 80% in group I, II, III, IV, V, VI, VII and less than 20% in group VIII were selected. This selection was to assure that the candidate proteins are specific for nephropathy due to T2DM as a triggering agent. Which implies to the fact that the proteins considered for further analysis were those thus, the protein bands obtained due to sharp peaks were further considered for the study. Further, maximum patients showing a similar protein profile were selected for further analysis. Patients with different protein excretion pattern and less in number were not considered (Fig. 1a–h).

Out of 48, only 35 normal healthy adults (NHA) showed a similar urinary protein pattern. Further, total 159 T2DM patients without any secondary complications were distributed into four different groups depending upon the duration of diabetes. Wherein, 37 out of 59 patients with 0–5 years T2DM duration showed protein pattern analogous to that of the NHA group. We observed that proteins of molecular weight 13, 17, 23, 24, 28, 35, 37, 39, 41, 49, 50 kDa were found significant in rest of the T2DM (Table 2) groups. Further, these proteins were found to increase gradually in 33 out of 38 patients of T2DM with 5–10 years of duration, 20 out of 37 patients with 10–15 years of T2DM and 19 out of 25 T2DM patients with 15–20 years of diabetes duration (Table 2) (p < 0.001). 39 out of 50 MIA patients and 69 out of 83 DN patients also showed a significant increase in the above-mentioned proteins (Table 2) (p < 0.001). 16 out of 50 patients from NDN group showed non-significant excretion of the LMWPs found in other T2DM groups (Table 2). However, NDN showed either lower or no concentration of the respective proteins (data of 260 one dimensional urinary profile is provided in Additional file 1). Thus, patients with such low molecular weight proteinuria were further selected for 2-dimensional electrophoresis study.

**Table 2:** Concentration of low molecular weight urinary proteins in T2DM patients with and without secondary complications

Molecular weight in kDa	Control (mg/dl)	T2DM with 0–5 years (mg/dl)	T2DM with 5–10 years (mg/dl)	T2DM with 10–15 years (mg/dl)	T2DM with 15–20 years (mg/dl)	Micro albuminuria (mg/dl)	Diabetic nephropathy (mg/dl)	Non diabetic nephropathy (mg/dl)
13	1.5	3.01 ^NS^a	12.37 ***ab	18.11 ***abc	26.82 ***abcd	99.93 ***abc,^NS^e	318.54 ***abcdef	0.67 ^NS^abcdefg
17	1.32	34.38 ***a	32.89 ***a^NS^b	32.62 ***a^NS^bc	37.64 ***a^NS^bcd	175.92 ***abcde	217.48 **abcdef	0 ^NS^abcdefg
23	15.42	15.42 ^NS^a	16.76 ^NS^ab	17.81 ^NS^abc	33.87 *a^NS^bcd	76.15 ***abcd **e	158.39 *abcdef	0.32 ^NS^abcdefg
24	2.42	5.42 ^NS^a	7.69 ^NS^ab	7.88 ^NS^abc	12.7 ***a *bcd	51.29 ***abcde	71.32 ***abcdef	0 ^NS^abcdefg
28	7.21	7.21 ^Ns^a	7.56 ^NS^ab	9.63 ^NS^abc	13.28 *abc^NS^d	109.35 ***abcde	71.83 ***abcdef	0.11 ^Ns^abcdefg
35	1.94	3.65 ^Ns^a	4.21 ^NS^ab	5.6 ^NS^abc	6.44 ^NS^abcd	45.68 ***abcde	52.09 ***abcdef	0 ^Ns^abcdefg
37	1.25	7.52 *a	7.86 *a^NS^b	8.21 *a^NS^bc	12.23 ***a *bcd	45.73 ***abcde	69.43 ***abcdef	0 ^NS^abcdefg
39	0.09	2.11 ^NS^a	2.2 ^NS^ab	2.45 ^NS^abc	3.05 ^NS^abcd	28.97 ***abcde	41.83 ***abcdef	0 ^NS^abcdefg
41	8.48	22.15 **a	23.14 **a^NS^b	26.71 **a^NS^bc	26.24 **a^NS^bcd	149.25 ***abcde	246.93 ***abcdef	0 ^NS^abcdefg
49	11.28	23.61 *a	23 *a^NS^b	23.57 *a^NS^bc	32.76 **a *bcd	214.62 ***abcde	341.59 **abcdef	0.14 ^NS^abcdefg
50	8.72	31.03 **a	31.5 **a^NS^b	32.6 **a^NS^bc	33.02 **a^NS^bcd	172.18 ***abcde	216.61 **abcdef	0 ^NS^abcdefg

The results are expressed as mean + SE and p < 0.05 is considered significant. * p < 0.05, ** p < 0.001, *** p < 0.0001

a—compared with control, b—compared with 0–5 years, T2DM, c—compared with 5–10 years T2DM, d—compared with 10–15 years T2DM, e—compared with 15–20 years T2DM, f—compared with microalbuminuria, g—compared with diabetic nephropathy

NS, non significant

### Identification of isolated proteins by 2D-PAGE

**Fig. 2:**
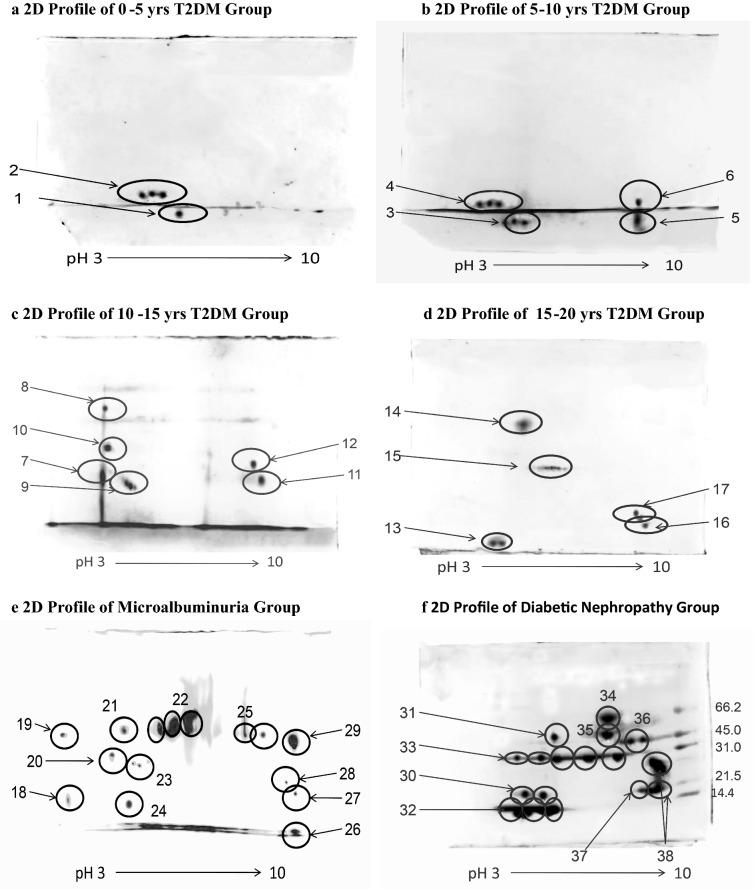
2 dimensional urinary proteomic profile of various study groups. 2 dimensional proteome map of different study group. As the duration of T2DM increases the excretion of low molecular weight protein also increases. The proteins were identified with MALDI QTOF. Upregulation of the low molecular weight proteins can be observed as the participants progresses to macroalbuminuria

The study predicted a significant set of proteins that are differentially expressed in the urine of T2DM patients with varying duration of diabetes (ranging from 1 to 20 years), microalbuminuria and diabetic nephropathy. As shown in Fig. 2a–f, total 25 areas from the 2-D gels representing single or multiple spots corresponding to the same protein were picked for further analysis. Analysis of MS/MS spectra by MS Spectrum Mill, de novo sequencing algorithms and peptide mass fingerprinting by MASCOT Analysis was carried on. This analysis includes mass by charge ratio and the probability based on MOWSE score (i.e. Molecular Weight SEarch) which helps to identify the protein. Peptides with the MOWSE score > 85 were selected, and 186 peptides were further aligned with Clustal Omega software. The calculation of molecular weight and pI of the probable protein was performed with ProtParam ExPASY tool. These 25 spots corresponded to total 13 different proteins. The details are given in Table 3. Post-translational modification were studied with GlycoMod and FindMod tools of ExPASY database.

**Table 3:** Urinary proteins identified with MALDI-QTOF

Spot no	Accession no	Protein name	MW (kDa)	pI	Protein/peptide excreted during	Classification of LMWPs depending upon its early excretion
1, 3, 9	P02766	Transthyretin/prealbumin	15.88	5.69	0–5, 5–10 and 10–15 years of T2DM	Group 1: LMWPs excreting in early years i.e. 0–5 and 5–10 years T2DM
2, 4, 13, 24, 32	P01834	Ig kappa C chain region	16.60	5.58	0–5, 5–10, 15–20 years of T2DM, MIA and DN	
5, 11, 16, 26, 27, 37	P01034	Cystatin C	15.79	9.00	5–10, 10–15, 15–20 years of T2DM, MIA and DN	
6, 12, 17, 28, 29, 38	P62979	Ubiquitin	17.96	9.00	5–10, 10–15, 15–20 years of T2DM, MIA and DN	
7, 8, 18, 19	P02763	Alpha 1 acid glycoprotein 1	23.51	4.93	10–15 years of T2DM and MIA	Group 2: LMWPs excreting in later years i.e. 10–15 and 15–20 years T2DM
10	P02647	Apolipoprotein A1	30.77	5.56	10–15 years of T2DM	
14	P25311	Zinc alpha 2 glycoprotein	37.25	5.71	15–20 years of T2DM	
15, 33	P02760	Alpha 1 microglobulin/bikunin precursor	35.99	5.95	15–20 years of T2DM and DN	
20, 21	P06727	Apolipoprotein A IV	45.39	5.28	MIA	Group 3: LMWPs excreting in advanced stages i.e. MIA and DN
22, 23, 31	P01009	Alpha 1 antitrypsin	46.73	5.37	MIA, DN	
25	P00738	Haptoglobin	45.20	6.13	MIA	
30	P01614	Ig kappa chain V–II	12.67	5.28	DN	
34, 35	Q86WR0	Protein 25	24.47	6.34	DN	
36	P0CG05	Ig lambda 2 chain region	11.29	6.91	DN	

Proteins were recognized using NCBI and SWISS PROT database of urine. The molecular weight and pI of the protein were calculated using ProtParam ExPASY tool

### Two-dimensional urinary protein profile

To avoid false positive estimation five patients with similar urinary 1 dimensional protein profile from each group were selected for further 2DE study. The 2DE of each selected participants were carried out twice. We found transthyretin/prealbumin and Ig kappa C chain region in 0–5 years of T2DM duration (Table 3, Fig. 2a). In T2DM patients with 5–10 years, we obtained transthyretin/prealbumin, Ig kappa C chain region, Cystatin, C, and Ubiquitin (Table 3, Fig. 2b). In 10–15 and 15–20 years of T2DM duration, we observed the appearance of Alpha 1 acid glycoprotein 1, Apolipoprotein A1, transthyretin/prealbumin, Ig kappa C chain region, AMBP, Pigment epithelium-derived factor, Cystatin C, Zinc Alpha 2 glycoprotein and Ubiquitin (Table 3, Fig. 2c, d). Further, microalbuminuria and diabetic nephropathy patients marked the excretion of Ig kappa chain V–II, protein 25, Ig lambda 2 chain region, Alpha 1 acid glycoprotein 1, Apolipoprotein A1, transthyretin/prealbumin, Ig kappa C chain region, AMBP, Cystatin C, zinc alpha 2 glycoprotein and Ubiquitin (Table 3, Fig. 2e, f). The above-mentioned proteins are significantly excreted in urine, whereas, serum creatinine, microalbumin, and eGFR were still within normal range. The proteins were involved in cell development, cell organization, defense response, metabolism, and signal transduction.

We have categorized the LMWPs into three different groups depending upon their excretion during the time interval of T2DM. Four proteinsincluded in Group 1 namely, Transthyretin/prealbumin, Ig kappa C chain region, Cystatin C and Ubiquitin excreted in early years (0–5 years) of T2DM and significantly expressed till DN. Amongst which transthyretin and Ig kappa chains excreted as early as 0–5 years of T2DM. However, transthyretin is not expressed after 10–15 years of T2DM. Ig kappa chains excretes through out the course of DN, right form 0 to 5 years T2DM up to DN. Cystatin c and Ubiquitin also excretes right form 0 to 5 years T2DM up to DN. From our study we can interpret that these four proteins either individually or in combination can prove as potential low molecular weight candidates capable of diagnosing early diabetic nephropathy. Group 2 includes Alpha1 acid glycoprotein1, Apolipoprotein A1, Zinc alpha 2 glycoprotein, Alpha 1 Microglobulin/Bikunin Precursor appear in late years of T2DM (at least in our patients) i.e. 10–15 and 15–20 years. Finally, Group 3 includes Apolipoprotein A IV, Alpha 1 antitrypsin, Haptoglobin, Ig kappa chain V–II, Protein 25, Ig lambda 2 chain region appear after the appearance of microalbumin and hence they are over expressed in patients with MIA and DN.

## Discussion

The most significant advantage of urinary proteomics is the prospect of a non-invasive and easy sampling system of diagnosis. Amongst the urinary proteome, 70% of the urinary proteins are of renal origin, and 30% are of systemic origin [4]. Thus, urinary proteomics may reduce the need for renal biopsy as the principal method for the diagnosis of the renal disorder. One of the major focus of our study was to establish a proteome map of low molecular weight proteinuria < 50 kDa with the help of 2DE in T2DM patients with/without complications and varying duration as well as chronicity of diabetes.

From one-dimensional protein profiling, we were able to screen total 11 different LMWPs with molecular weight 13, 17, 23, 24, 28, 35, 37, 39, 41, 49 and 50 kDa significantly predicting early DN. However, Visith et al. on multiple comparisons revealed nine spectra corresponding to 9 different individual bands (proteins ranging from 3.0 to 31.0 kDa) which significantly differentiated diabetic nephropathy from normal [8]. This variation may be due to the difference in sample size and study population. Our study includes four T2DM groups with varying duration of diabetes (ranging from 1 to 20 years) without any other secondary complications. T2DM patients with 0–5 years of diabetes duration showed protein profile analogous to that of the NHA group with an insignificant excretion of proteins. We can thus predict that the cutoff value of T2DM duration for the onset of renal alterations is about 5 years which resemble the observation reported by Rao et al. [9]. We for the first time reported a notable appearance of LMWPs (< 50 kDa) in the patients with 5–10 years of T2DM duration which can be attributed to protein overload or inefficiency of proximal tubules to reabsorb LMWPs [10]. We observed the presence of LMWPs in T2DM patients with normal renal functioning (serum creatinine and microalbuminuria still within normal ranges) indicating normal glomerular filtration and minimal proteinuria. This is indicative of tubular proteinuria/protein overload phase before the appearance of macroalbuminuria from where the condition can be reverted or ceased to worsen further.

There are many studies showing the utility of 2DE for establishing proteome map useful in differentiating DN patients from control groups [11–13]. But we for the first time screened excretion of proteins depending upon the duration of T2DM. From our observations, we found total eight LMWPs (Table 3; Group 1 and 2) capable of predicting the earliest subclinical renal alterations when serum creatinine, eGFR, and microalbuminuria are within normal range. From Group 1, Ig kappa light chains (KLC) showed their excretion as early as 0–5 years of T2DM which even Groop et al. had reported that T2DM subjects (type 1 and type 2 both) with short duration (1 year) and normoalbuminuria excreted KLC [14]. It was found that nonenzymatic glycation of KLC interferes with its normal tubular reabsorption leading to its direct excretion in urine [14]. In addition to this Hutchison et al. has also reported fivefold urinary KLC in patients with progressive renal injury [15]. A study reported by Zurbig et al. showed the importance of collagen fragments in early detection of DN [16]. Another LMWP, i.e., transthyretin/pre-albumin though excretes as early as 0–5 years of T2DM but surprisingly it was not expressed after 10–15 years of T2DM. However, this observation was not in accordance with Bellei et al. who found transthyretin to be progressively increase from T2DM patients with normoalbuminuria to DN [12], whereas, on the other hand, Rao et al. found it to be downregulating [11].

Excretion of cystatin c and ubiquitin was increased in patients with the duration of 5–10 years T2DM. We for the first time showed the expression of cystatin c in urine. Cystatin c is reabsorbed and catabolized in proximal tubules and hence its diagnostic excretion is increased in proximal tubular injury [17]. Dihazi et al. reported a shorter form of ubiquitin protein (≈ 8 kDa) efficiently diagnosing DN subjects [18]. Oxidative and carbonyl stress regulates the excessive ubiquitin expression [19], whereas, its selective expression in the renal tubules suggests that the ubiquitin–proteasome proteolytic system is active in this compartment of the kidney and plays a vital role in the progression of DN [18]. But none of the study apart from us has mentioned the time-dependent appearance of LMWPs.

Hyperglycemia could lead to LMWPs excretion by affecting glomerular filtration and the tubular reabsorption of LMWPs. Christiansen et al. showed increased GFR due to moderate hyperglycemia in normal participants attained by intravenous glucose infusion [20]. In addition to this even glucose-induced osmotic diuresis could increase excretion of proximal tubule-derived LMWPs. There are several well known pathologic conditions related to proximal tubules responsible for the increase/decrease in LMWPs. The rate of protein filtration at the glomerulus and its reabsorption at proximal tubules might not be to the same extent. Thus, it depicts that the renal handling of the individual protein is completely different. The excreted urinary LMWPs can reveal the mechanism of its appearance or disappearance. Proteins/peptides directly appear in urine only if they have avoided tubular reabsorption and secreted by renal cells or lower urinary tract. The structural and functional proximal tubular changes during the early course of DN lead to the increased excretion of LMWPs > 40 kDa [10]. This implies that proximal tubular damage in T2DM subjects plays a vital role in progression to macroalbuminuria and increased LMWP excretion may imply renal tubular disorder in T2DM.

Jain et al. found 3 LMWPs namely Alpha 1 Microglobulin/Bikunin Precursor (A1M), Alpha1 acid glycoprotein1 (A1AG1) and Zinc alpha 2 glycoprotein (ZA2G) capable of discriminating diabetic nephropathy from control subjects but they could not recognize the precise stage of renal injury these LMWPs were excreted [21], whereas, we observed A1AG1, Apolipoprotein A1 (ApA1), ZA2G and A1M appeared in late years of T2DM, i.e., 10–15 and 15–20 years. A1AG1 is a well-established marker of inflammation and has been reported to predict mortality in T2DM subjects [22]. Even Rao et al. reported upregulation of ZA2G levels and it is the possible candidate for regulation body weight [11, 20]. A1M levels were significantly correlated to the duration and progression of T2DM [23]. They reported that A1M levels were highest amongst the subjects on insulin treatment which correlated with our observations, where we found that subjects with 15–20 years of T2DM duration (late years of T2DM) also were dependent more on insulin treatment for controlling hyperglycemia (≈ 30.8% subjects) in comparison to other T2DM patients w/o secondary complications.

Apolipoprotein A IV (Apo A4), Alpha 1 antitrypsin (A1AT), Haptoglobin (Hp), Ig kappa chain V–II/Ig lambda 2 chain (Ig chains) and Protein 25 appear in well-advanced stages. Apo A4 is significantly correlated with subjects having macular edema [24]. A1AT was first reported by Rao et al. to be upregulated [11] and Ig chains has a vital role in inflammation. Hp is a transport protein, and Hp allele 2 has a vital role in the development of DN [25]. The role of protein 25 is not well defined, but it is a predicted substrate of insulin receptor ad has a vital role in cell signaling. Its expression is ubiquitous in tissues and is released in urine due to tissue damage.

Megalin and cubulin are two endocytic receptors which both together facilitate reabsorption of almost all LMWPs, including albumin [26]. However, the affinity of RBP, β-2 microglobulin, NGAL and Vitamin D-binding protein to megalin is very low which indicates that their urinary excretion should increase along with the progression of albuminuria. Surprisingly we did not observe excretion of any above-mentioned such LMWPs in our study groups. These observations infer that an increase in urinary excretion of LMWPs is dependent on the degree to which the LMWPs compete for reabsorption in comparison to albumin. Even Groop et al. due to similar reason found increased urinary excretion of KLC (23 kDa) rather than β-2 microglobulin (12 kDa) [14]. Since, the LMWPs concentrations are affected by both competition for absorption and tubular injury [27]. Even, Thielemans et al. demonstrated the ability of albumin to inhibit reabsorption of certain LMWPs indicative of competition for a common transport mechanism [28]. Excretion of all the above-mentioned LMWPs indicates that their affinity for megalin-cubulin is decreased. Overall the urinary concentration of individual LMWP is dependent upon (1) filtration rate of LMWPs at the glomerulus, (2) its reabsorption rate at proximal tubule and (3) competition for absorption by albumin.

We also noted a difference in 2D patterns of urinary proteins in different groups of patients (Fig. 2). Variations in such 2D patterns are due to the difference in the physiological processes like glomerular filtration, tubular reabsorption of the proteins, blood pressure, renal blood flow, glomerular capillary pressure and blood levels of vasoactive peptides or hormones [13]. Interestingly, T2DM patients with normoalbuminuria gave a different protein pattern in comparison to T2DM patients with MIA and DN. The position of similar proteins like Transthyretin and IgG kappa chain C region was shifted in the case of normoalbuminuric and macroalbuminuric T2DM patients. We observed that the molecular weight and pI varied from its theoretical molecular weight. Lafitte et al. also reported the similar difference between actual observed and theoretical molecular weight of protein [13]. This discrepancy might be due to post-translational modifications like glycosylation/phosphorylation [29]. An additional reason might be the low buffering capacity of small proteins, which are unable to migrate at their original isoelectric point [30].

Though acetone precipitation is one of the best technique for isolation of LMWPs, as it can recover the widest range of LMWPs from urine [31]. We observed that protein of molecular weight less than 11 kDa were not isolated. This might be due to protein size plays a critical role in its precipitation by acetone, as the solvent concentration required for precipitation is inversely proportional to protein size [32]. Smaller proteins are more difficult to precipitate because their little-charged surface area produces less repulsive [32].

## Conclusion

We have assessed urinary proteins with molecular weight < 50 kDa by 2-D gel electrophoresis and MALDI Q-TOF and found a small number of proteins that were upregulated at the different time interval of T2DM and their levels increased along with the increasing degree of albuminuria. These proteins were exclusive for T2DM and proteins with significant excretion in nondiabetic nephropathies were excluded from the study. This makes the current study unique amongst the past studies carried out till date. To determine the prospective efficacy of the candidate proteins we have included a well-defined and well-characterized patient population. The participants included are specifically age-matched, biochemical profile, matched and belonged to similar diabetes duration. Thus, this will not detract the limitation of the present experiment which is cross-sectional and with a restricted number of participants in different study groups. Regardless of this, a longitudinal study is required for validating the efficiency of the suggested proteomic pattern. Studying the patterns at regular interval for certain duration of time will help to infer the rate of progression of disease and closely monitor the patients for the same. We also accept the fact that the conventional methods like HbA1C and urinary dipstick methods help to monitor the disease and are by far the gold standard methods, but one cannot deny the fact that they are the markers of well established disease. The dipstick method can conventionally diagnose proteinuria at 300 mg/day and HbA1C can predict well established diabetes. However, our present study can provide a better and early prediction to the insight of such slow progressive disease and can work better than HbA1C and the traditional urinary dipstick method. The data finally suggested the utility of proteomic analysis be useful for detection of low abundant proteins. We have identified eight different proteins present in T2DM patients (with and without complications) which can be further characterized by ELISA or western blotting to confirm their utility in detecting early diabetic nephropathy. Out of which four proteins (Ig light chains, transthyretin, cystatin c and ubiquitin) either individually or in combination can prove as potential low molecular weight candidates capable of diagnosing early diabetic nephropathy as early as 0–5 years of T2DM and 5–10 years of T2DM but their validation in large number of population is to be verified.

## Additional file

**Additional file 1.** Urinary protein electeropherograms of control groups, participants with varrying duration of diabetes with normal kidney functioning, microalbuminuria and nephropathy.
