# Supplementary material for: Characterization of low molecular weight urinary proteins at varying time intervals in type 2 diabetes mellitus and diabetic nephropathy patients
Source: Diabetol Metab Syndr. 2019 May 17;11:39. doi: 10.1186/s13098-019-0430-1 (PMC6525442; doi:10.1186/s13098-019-0430-1)
Supplement: Supplementary file 1 — Additional file 1. Urinary protein electeropherograms of control groups, participants with varrying duration of diabetes with normal kidney functioning, microalbuminuria and nephropathy. [file 13098_2019_430_MOESM1_ESM.pptx]

## Slide 1
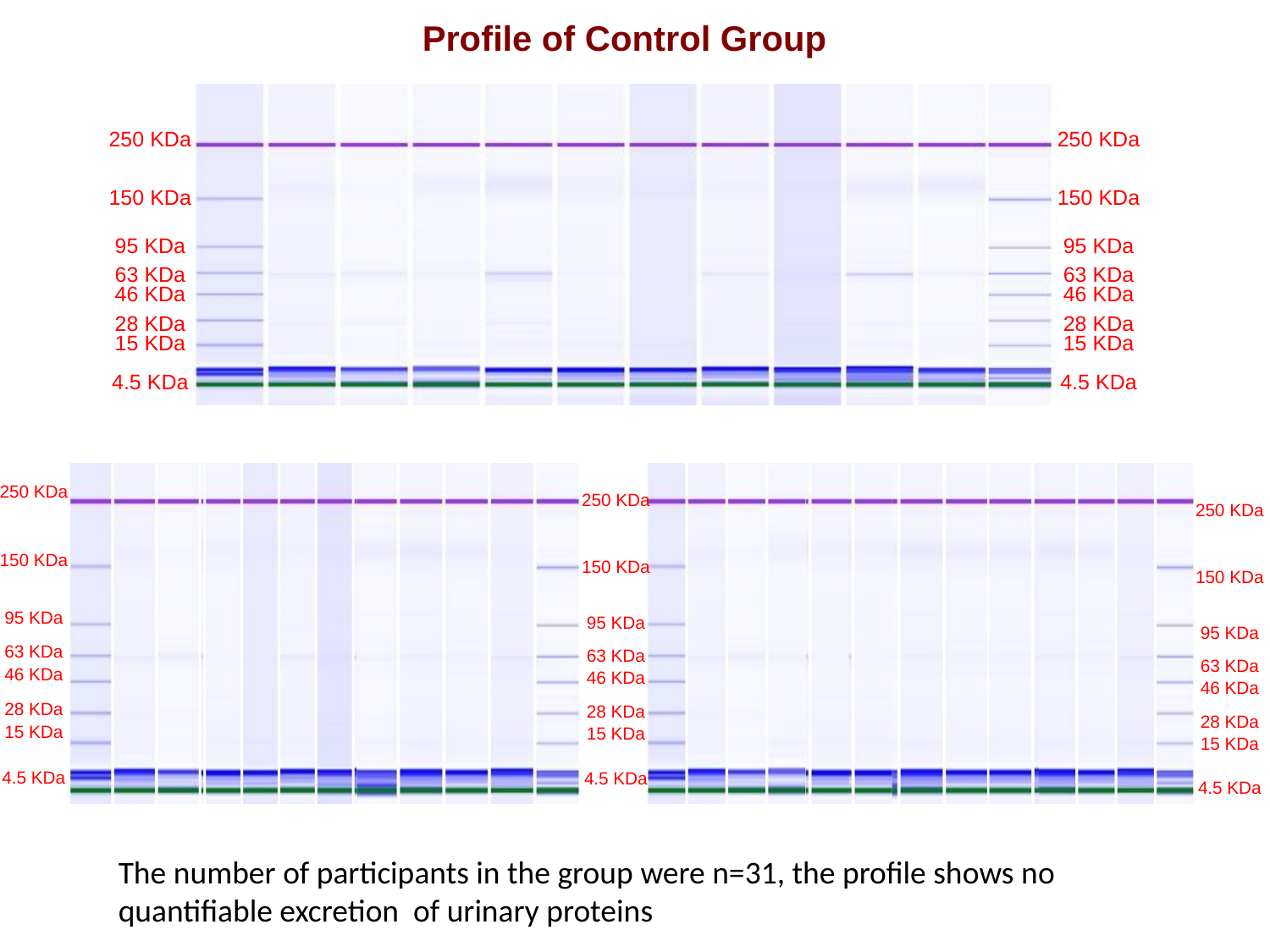

Profile of Control Group
250 KDa
250 KDa
150 KDa
150 KDa
95 KDa
95 KDa
63 KDa
63 KDa
46 KDa
46 KDa
28 KDa
28 KDa
15 KDa
15 KDa
4.5 KDa
4.5 KDa
250 KDa
150 KDa
95 KDa
63 KDa
46 KDa
28 KDa
15 KDa
4.5 KDa
250 KDa
150 KDa
95 KDa
63 KDa
46 KDa
28 KDa
15 KDa
4.5 KDa
250 KDa
150 KDa
95 KDa
63 KDa
46 KDa
28 KDa
15 KDa
4.5 KDa
The number of participants in the group were n=31, the profile shows no quantifiable excretion of urinary proteins

## Slide 2
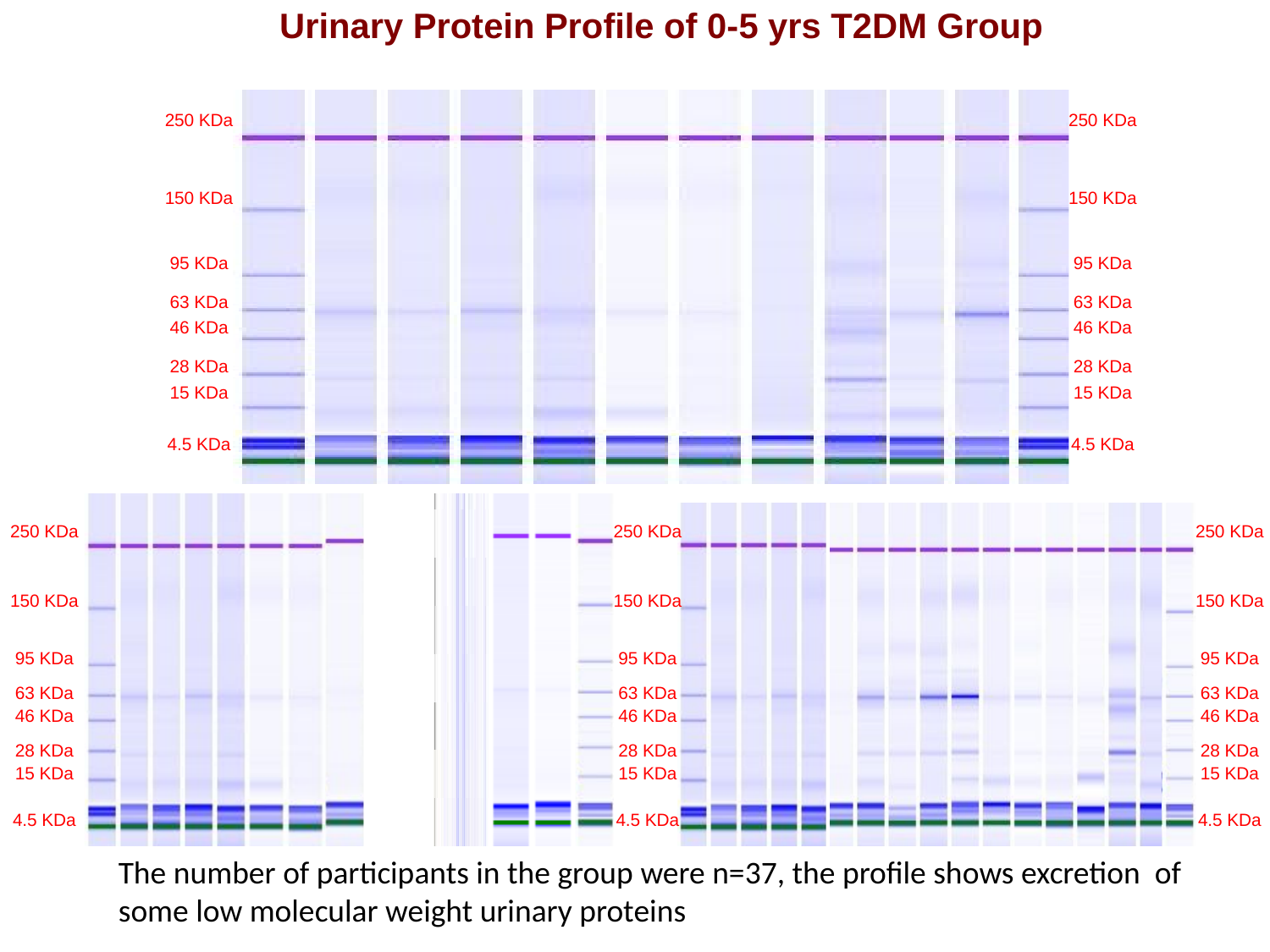

Urinary Protein Profile of 0-5 yrs T2DM Group
250 KDa
150 KDa
95 KDa
63 KDa
46 KDa
28 KDa
15 KDa
4.5 KDa
250 KDa
150 KDa
95 KDa
63 KDa
46 KDa
28 KDa
15 KDa
4.5 KDa
250 KDa
150 KDa
95 KDa
63 KDa
46 KDa
28 KDa
15 KDa
4.5 KDa
250 KDa
150 KDa
95 KDa
63 KDa
46 KDa
28 KDa
15 KDa
4.5 KDa
250 KDa
150 KDa
95 KDa
63 KDa
46 KDa
28 KDa
15 KDa
4.5 KDa
The number of participants in the group were n=37, the profile shows excretion of some low molecular weight urinary proteins

## Slide 3
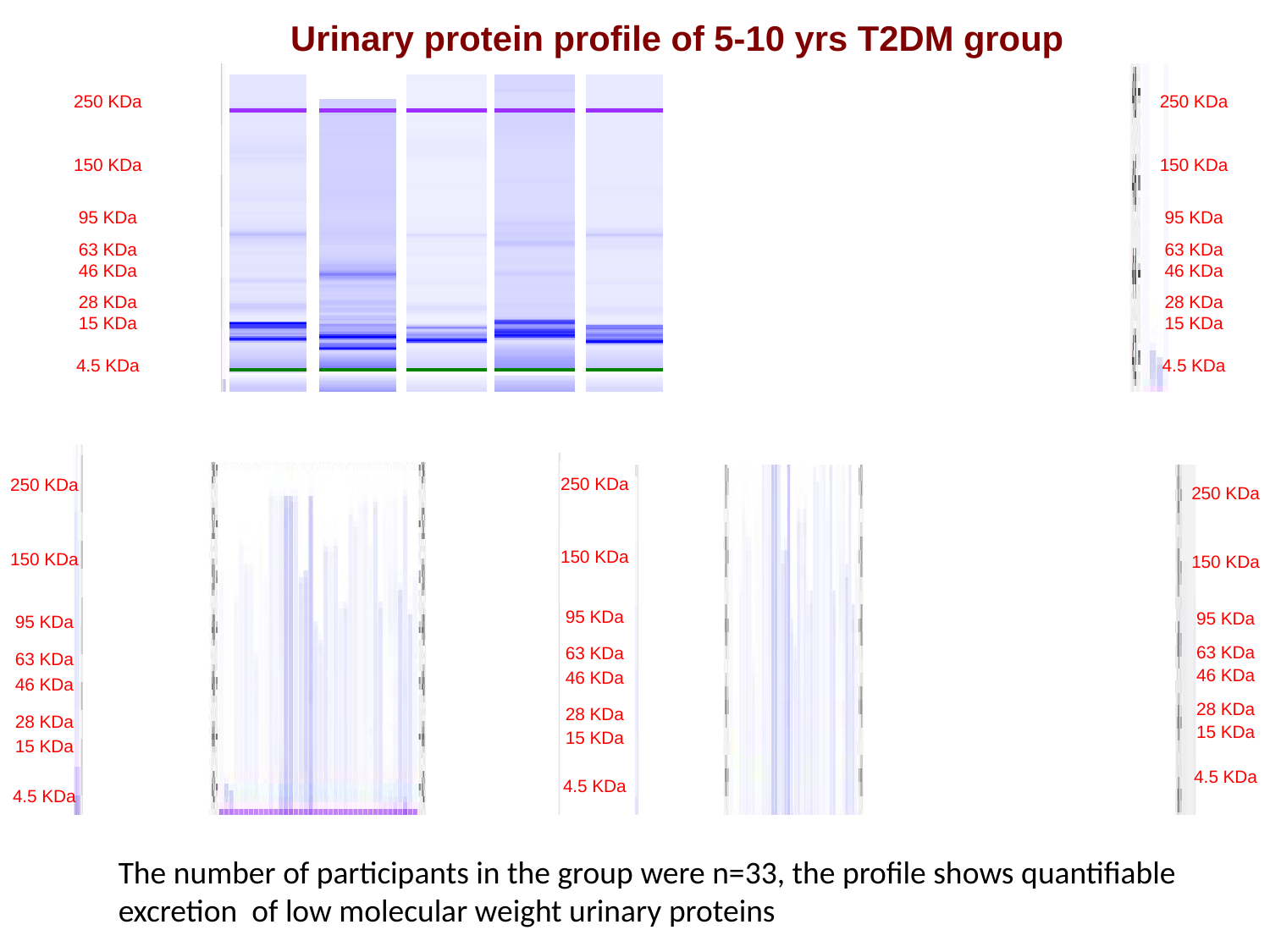

Urinary protein profile of 5-10 yrs T2DM group
250 KDa
150 KDa
95 KDa
63 KDa
46 KDa
28 KDa
15 KDa
4.5 KDa
250 KDa
150 KDa
95 KDa
63 KDa
46 KDa
28 KDa
15 KDa
4.5 KDa
250 KDa
150 KDa
95 KDa
63 KDa
46 KDa
28 KDa
15 KDa
4.5 KDa
250 KDa
150 KDa
95 KDa
63 KDa
46 KDa
28 KDa
15 KDa
4.5 KDa
250 KDa
150 KDa
95 KDa
63 KDa
46 KDa
28 KDa
15 KDa
4.5 KDa
The number of participants in the group were n=33, the profile shows quantifiable excretion of low molecular weight urinary proteins

## Slide 4
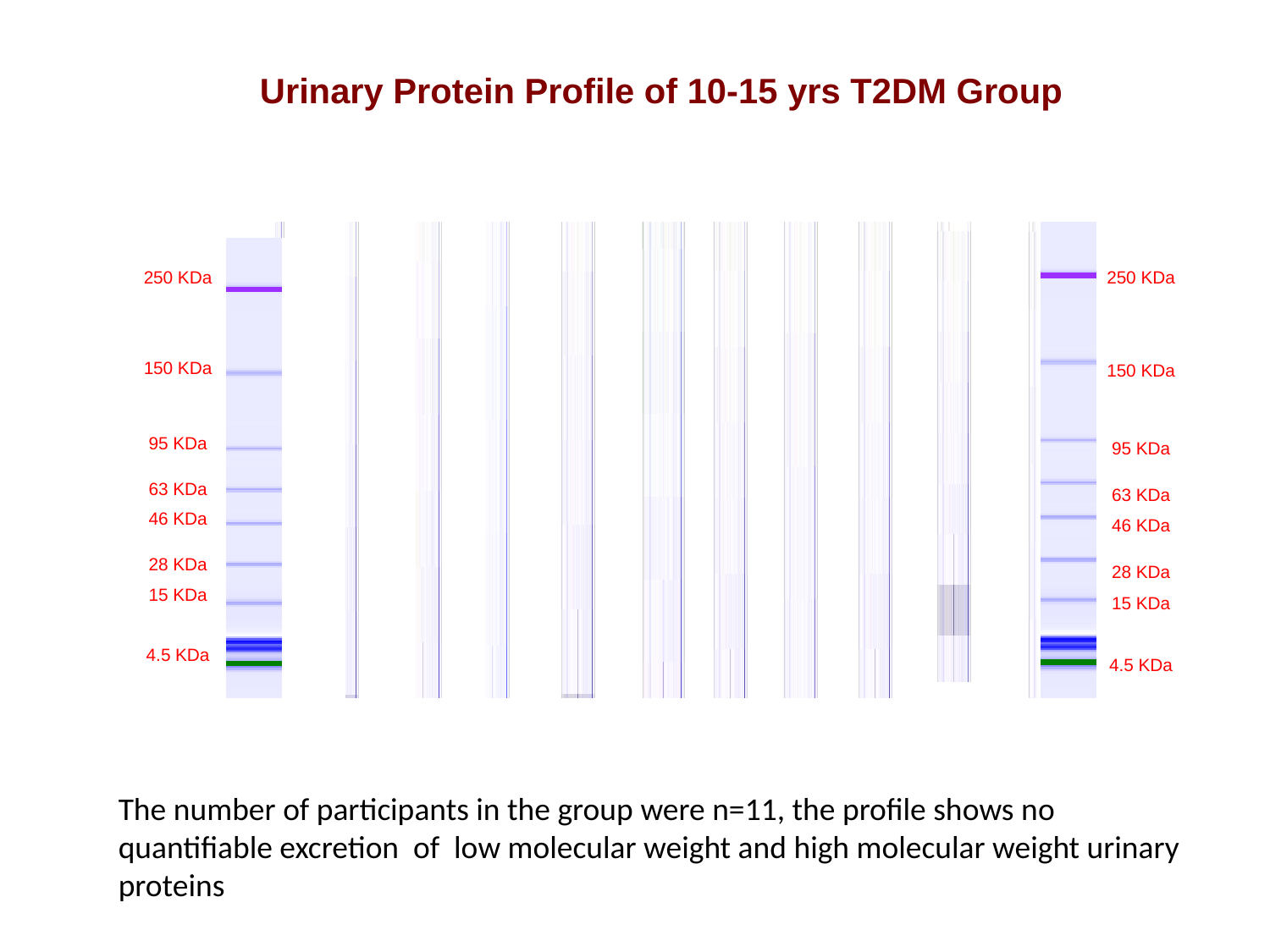

Urinary Protein Profile of 10-15 yrs T2DM Group
250 KDa
150 KDa
95 KDa
63 KDa
46 KDa
28 KDa
15 KDa
4.5 KDa
250 KDa
150 KDa
95 KDa
63 KDa
46 KDa
28 KDa
15 KDa
4.5 KDa
The number of participants in the group were n=11, the profile shows no quantifiable excretion of low molecular weight and high molecular weight urinary proteins

## Slide 5
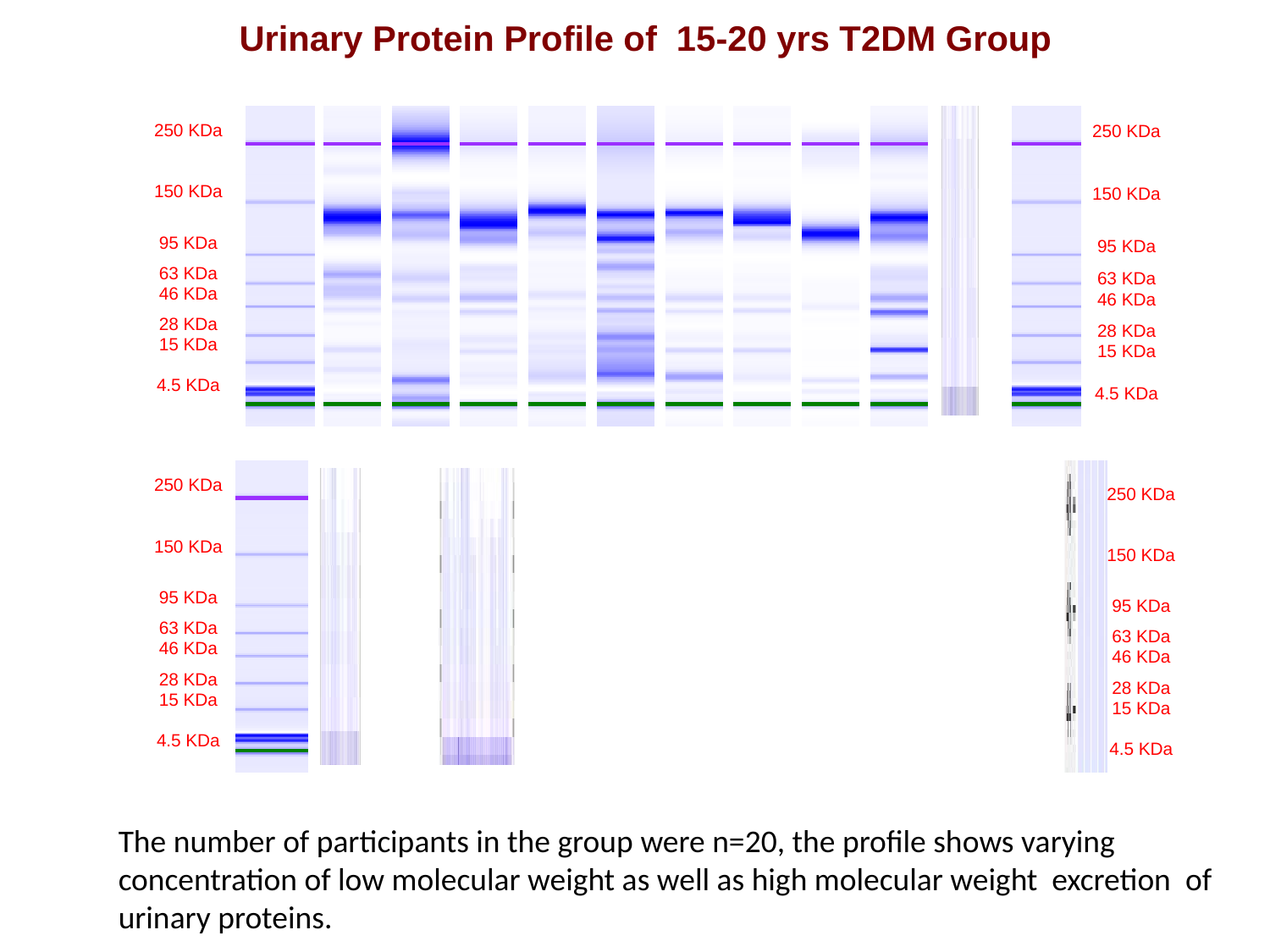

Urinary Protein Profile of 15-20 yrs T2DM Group
250 KDa
150 KDa
95 KDa
63 KDa
46 KDa
28 KDa
15 KDa
4.5 KDa
250 KDa
150 KDa
95 KDa
63 KDa
46 KDa
28 KDa
15 KDa
4.5 KDa
250 KDa
150 KDa
95 KDa
63 KDa
46 KDa
28 KDa
15 KDa
4.5 KDa
250 KDa
150 KDa
95 KDa
63 KDa
46 KDa
28 KDa
15 KDa
4.5 KDa
The number of participants in the group were n=20, the profile shows varying concentration of low molecular weight as well as high molecular weight excretion of urinary proteins.

## Slide 6
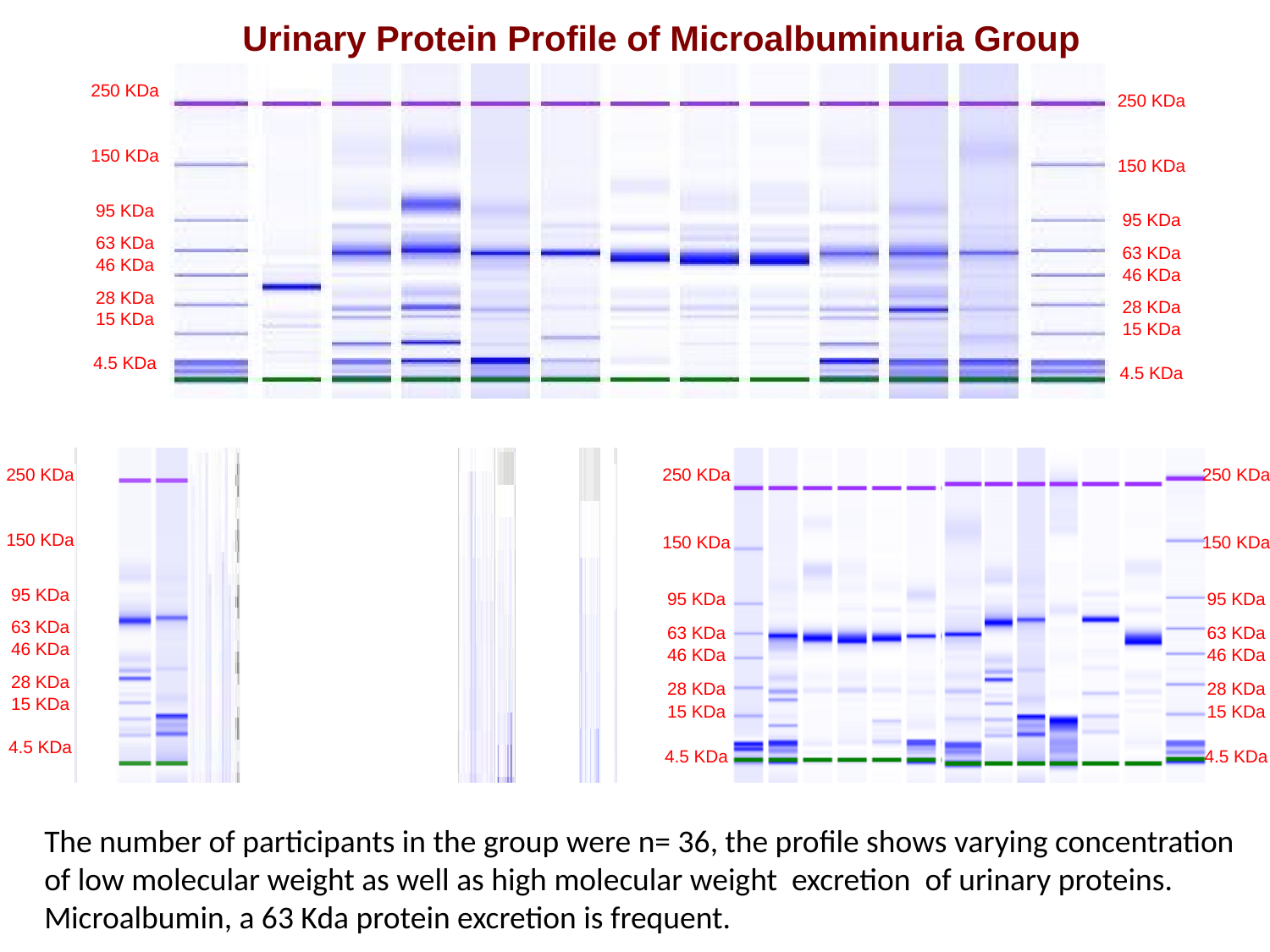

Urinary Protein Profile of Microalbuminuria Group
250 KDa
150 KDa
95 KDa
63 KDa
46 KDa
28 KDa
15 KDa
4.5 KDa
250 KDa
150 KDa
95 KDa
63 KDa
46 KDa
28 KDa
15 KDa
4.5 KDa
250 KDa
150 KDa
95 KDa
63 KDa
46 KDa
28 KDa
15 KDa
4.5 KDa
250 KDa
150 KDa
95 KDa
63 KDa
46 KDa
28 KDa
15 KDa
4.5 KDa
250 KDa
150 KDa
95 KDa
63 KDa
46 KDa
28 KDa
15 KDa
4.5 KDa
The number of participants in the group were n= 36, the profile shows varying concentration of low molecular weight as well as high molecular weight excretion of urinary proteins. Microalbumin, a 63 Kda protein excretion is frequent.

## Slide 7
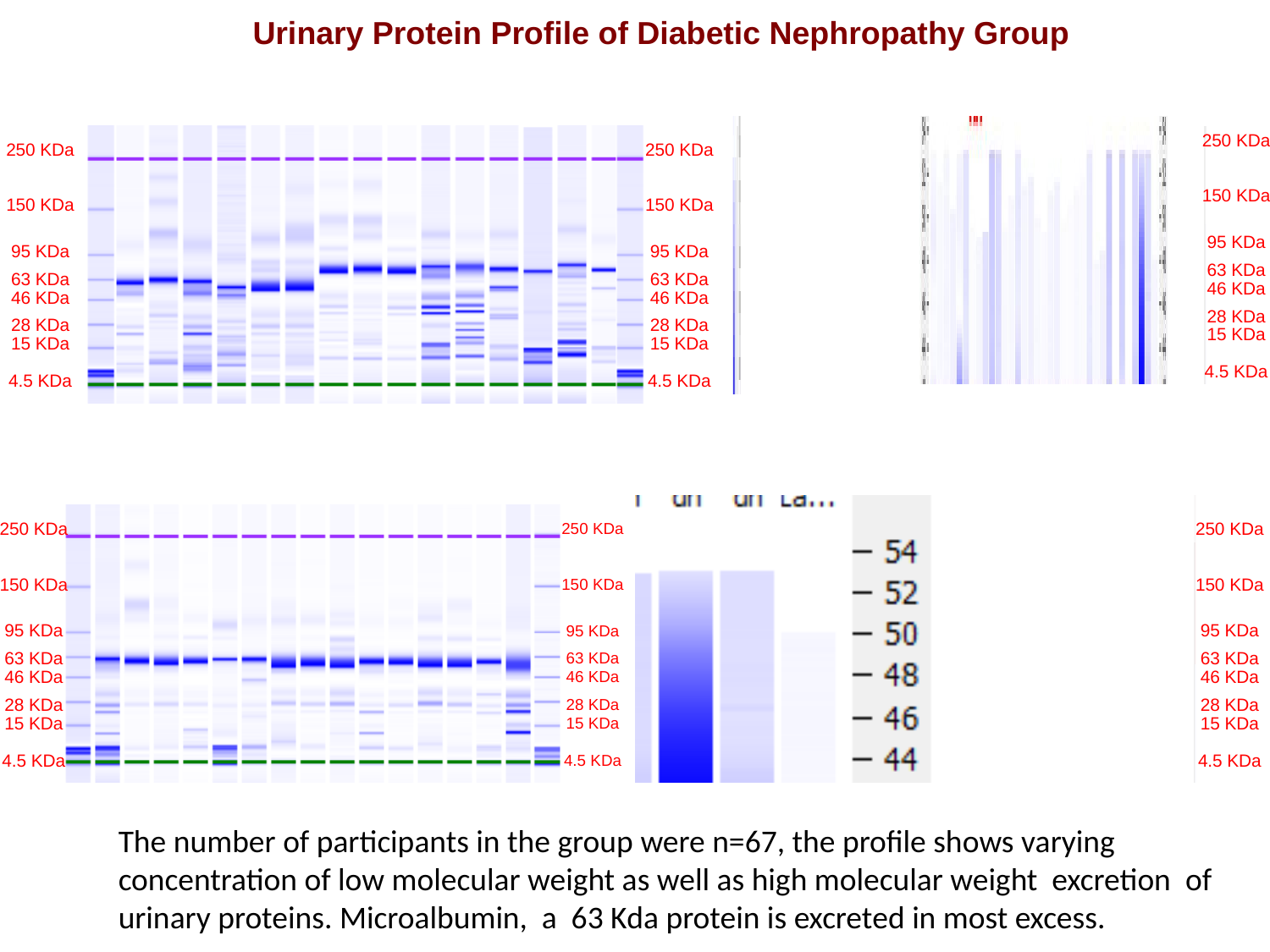

Urinary Protein Profile of Diabetic Nephropathy Group
250 KDa
150 KDa
95 KDa
63 KDa
46 KDa
28 KDa
15 KDa
4.5 KDa
250 KDa
150 KDa
95 KDa
63 KDa
46 KDa
28 KDa
15 KDa
4.5 KDa
250 KDa
150 KDa
95 KDa
63 KDa
46 KDa
28 KDa
15 KDa
4.5 KDa
250 KDa
150 KDa
95 KDa
63 KDa
46 KDa
28 KDa
15 KDa
4.5 KDa
250 KDa
150 KDa
95 KDa
63 KDa
46 KDa
28 KDa
15 KDa
4.5 KDa
250 KDa
150 KDa
95 KDa
63 KDa
46 KDa
28 KDa
15 KDa
4.5 KDa
The number of participants in the group were n=67, the profile shows varying concentration of low molecular weight as well as high molecular weight excretion of urinary proteins. Microalbumin, a 63 Kda protein is excreted in most excess.

## Slide 8
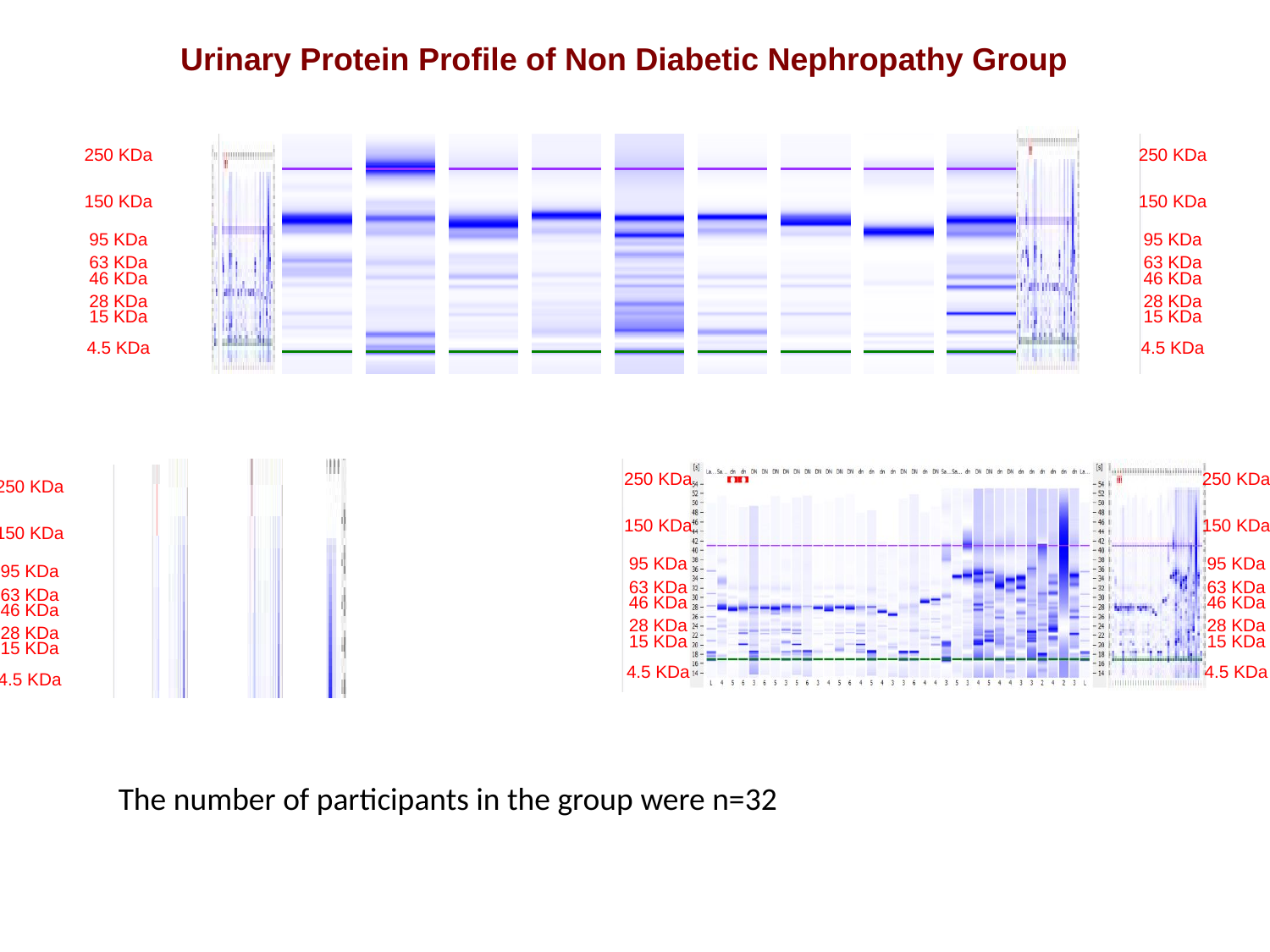

Urinary Protein Profile of Non Diabetic Nephropathy Group
250 KDa
150 KDa
95 KDa
63 KDa
46 KDa
28 KDa
15 KDa
4.5 KDa
250 KDa
150 KDa
95 KDa
63 KDa
46 KDa
28 KDa
15 KDa
4.5 KDa
250 KDa
150 KDa
95 KDa
63 KDa
46 KDa
28 KDa
15 KDa
4.5 KDa
250 KDa
150 KDa
95 KDa
63 KDa
46 KDa
28 KDa
15 KDa
4.5 KDa
250 KDa
150 KDa
95 KDa
63 KDa
46 KDa
28 KDa
15 KDa
4.5 KDa
The number of participants in the group were n=32
